# Airborne Bacteria in Earth's Lower Stratosphere Resemble Taxa Detected in the Troposphere: Results From a New NASA Aircraft Bioaerosol Collector (ABC)

**DOI:** 10.3389/fmicb.2018.01752

**Published:** 2018-08-14

**Authors:** David J. Smith, Jayamary Divya Ravichandar, Sunit Jain, Dale W. Griffin, Hongbin Yu, Qian Tan, James Thissen, Terry Lusby, Patrick Nicoll, Sarah Shedler, Paul Martinez, Alejandro Osorio, Jason Lechniak, Samuel Choi, Kayleen Sabino, Kathryn Iverson, Luisa Chan, Crystal Jaing, John McGrath

**Affiliations:** ^1^NASA Ames Research Center, Space Biosciences Division Moffett Field, CA, United States; ^2^Second Genome Inc. South San Francisco, CA, United States; ^3^United States Geological Survey, Environmental Health St. Petersburg, FL, United States; ^4^Climate and Radiation Laboratory, NASA Goddard Space Flight Center Greenbelt, MD, United States; ^5^Earth Science Division, Bay Area Environmental Research Institute Moffett Field, CA, United States; ^6^Lawrence Livermore National Laboratory Livermore, CA, United States; ^7^Space Biosciences Division, Blue Marble Space Institute of Science Moffett Field, CA, United States; ^8^Biological Oceanography Department, University of South Florida, College of Marine Sciences St. Petersburg, FL, United States; ^9^NASA Armstrong Flight Research Center Palmdale, CA, United States; ^10^Jacobs Technology Inc., NASA Armstrong Flight Research Center Palmdale, CA, United States

**Keywords:** bioaerosols, bacteria, C-20A, troposphere, stratosphere, Aircraft Bioaerosol Collector (ABC)

## Abstract

Airborne microorganisms in the upper troposphere and lower stratosphere remain elusive due to a lack of reliable sample collection systems. To address this problem, we designed, installed, and flight-validated a novel Aircraft Bioaerosol Collector (ABC) for NASA's C-20A that can make collections for microbiological research investigations up to altitudes of 13.7 km. Herein we report results from the first set of science flights—four consecutive missions flown over the United States (US) from 30 October to 2 November, 2017. To ascertain how the concentration of airborne bacteria changed across the tropopause, we collected air during aircraft *Ascent*/*Descent* (0.3 to 11 km), as well as sustained *Cruise* altitudes in the lower stratosphere (~12 km). Bioaerosols were captured on DNA-treated gelatinous filters inside a cascade air sampler, then analyzed with molecular and culture-based characterization. Several viable bacterial isolates were recovered from flight altitudes, including *Bacillus* sp., *Micrococcus* sp., *Arthrobacter* sp., and *Staphylococcus* sp. from Cruise samples and *Brachybacterium* sp. from Ascent/Descent samples. Using 16S V4 sequencing methods for a culture-independent analysis of bacteria, the average number of total OTUs was 305 for Cruise samples and 276 for Ascent/Descent samples. Some taxa were more abundant in the flight samples than the ground samples, including OTUs from families *Lachnospiraceae, Ruminococcaceae* and *Erysipelotrichaceae* as well as the following genera: *Clostridium, Mogibacterium, Corynebacterium, Bacteroides, Prevotella, Pseudomonas*, and *Parabacteroides*. Surprisingly, our results revealed a homogeneous distribution of bacteria in the atmosphere up to 12 km. The observation could be due to atmospheric conditions producing similar background aerosols across the western US, as suggested by modeled back trajectories and satellite measurements. However, the influence of aircraft-associated bacterial contaminants could not be fully eliminated and that background signal was reported throughout our dataset. Considering the tremendous engineering challenge of collecting biomass at extreme altitudes where contamination from flight hardware remains an ever-present issue, we note the utility of using the stratosphere as a proving ground for planned life detection missions across the solar system.

## Introduction

Microbial “highways” flow naturally overhead in Earth's atmosphere (Schmale and Ross, 2015) but “traffic patterns” elude the international aerobiology research community due to a widespread shortage of sampling opportunities. Although airborne biomass eventually returns to the surface, low sedimentation rates of microorganisms allows potentially long periods aloft in the upper atmosphere and, consequently, long distances traveled downwind (Bovallius et al., 2006; Reche et al., 2018). A variety of ground and airborne studies have recently reported that viable microorganisms can be delivered across continents and oceans (Prospero et al., 2005; Bowers et al., 2011; Toepfer et al., 2011; Favet et al., 2012; Smith et al., 2012, 2013; Yamaguchi et al., 2012; Barberán et al., 2015; Tang et al., 2016; Gat et al., 2017; Maki et al., 2017; Weil et al., 2017), microbes at lower altitudes in clouds might be temporarily active (Klein et al., 2016; Amato et al., 2017c), biomass (intact cells, spores, or debris) can influence cloud chemistry and precipitation patterns (Deguillaume et al., 2008; Bowers et al., 2009; Vaïtilingom et al., 2010, 2013; Amato et al., 2015), and biosignatures can still be detected up to 38 km (Smith, 2013). Since all marine and surface environments are impacted by winds, the emanation/deposition of airborne microorganisms (hereafter referred to as bioaerosols) contributes to ecosystem dynamics. For instance, airborne microorganisms surviving harsh conditions while aloft, including strong levels of mutagenic ultraviolet radiation, might be altered at the genomic, transcriptomic, and proteomic level upon germination in a new environment (Smith et al., 2011; Chudobova et al., 2015; Waters et al., 2015; Khodadad et al., 2017). With hundreds of teragrams of microbe-laden dusts from deserts and agricultural soils moving through Earth's atmosphere each year (Acosta-Martinez et al., 2015), additional surveys are needed to better understand the ecological consequences of airborne biomass exchange, including disease dispersal (Brown and Hovmoller, 2002; Fröhlich-Nowoisky et al., 2016; Mahaffee and Stoll, 2016; Van Leuken et al., 2016). Ultimately, more sampling opportunities above the boundary layer (i.e., >2 km above the Earth's surface) will improve long range modeling efforts aimed at providing predictive tools for aerobiology studies at regional and global scales (Burrows et al., 2009a,b; Griffin et al., 2017).

Before future investigations addressing the origin, destination, survival, and mutation of airborne microorganisms can be realistically implemented, routine and reliable access to the upper atmosphere for collecting bioaerosols must first be established. High-altitude aircraft are a compelling choice for capturing airborne microorganisms considering the multitude of government and commercially operated platforms flying into the upper atmosphere every day. Ride-along sample collections on aircraft would enable transformative opportunities for the aerobiology research community by providing substantial spatiotemporal coverage. Despite an abundance of aircraft worldwide, surprisingly few studies have attempted in-flight collections of bioaerosols, perhaps because no standard hardware package exists for sample acquisition. Detailed engineering schematics and discussions of contamination control techniques onboard aircraft are notably absent in the small group of aircraft-based aerobiology literature (Meier and Lindbergh, 1935; Polunin and Kelly, 1952; Timmons et al., 1966; Trägårdh, 1977; Borodulin et al., 2005; Hill et al., 2007; DeLeon-Rodriguez et al., 2013; Maki et al., 2013). As the sensitivity and affordability of molecular methods in microbiology improves each year, so should a general awareness that hardware used for aerobiology surveys can be highly susceptible to contaminants (Smith and Griffin, 2013; Griffin et al., 2017). In the era of molecular assays, the aerobiology research community must adopt stricter quality control procedures—reporting detailed hardware designs, contamination control approaches and monitoring methods—similar to standard practices in space exploration (e.g., life detection and planetary protection) (Vaishampayan et al., 2013; Benardini et al., 2014; Summons et al., 2014) and industry (e.g., pharamceutical manufacuturing facilities). Improvements evaluating contamination have been recently made in studies using high altitude balloons (Bryan et al., 2014) but such stringency has not yet been implemented with aircraft experiments.

**Figure 1:**
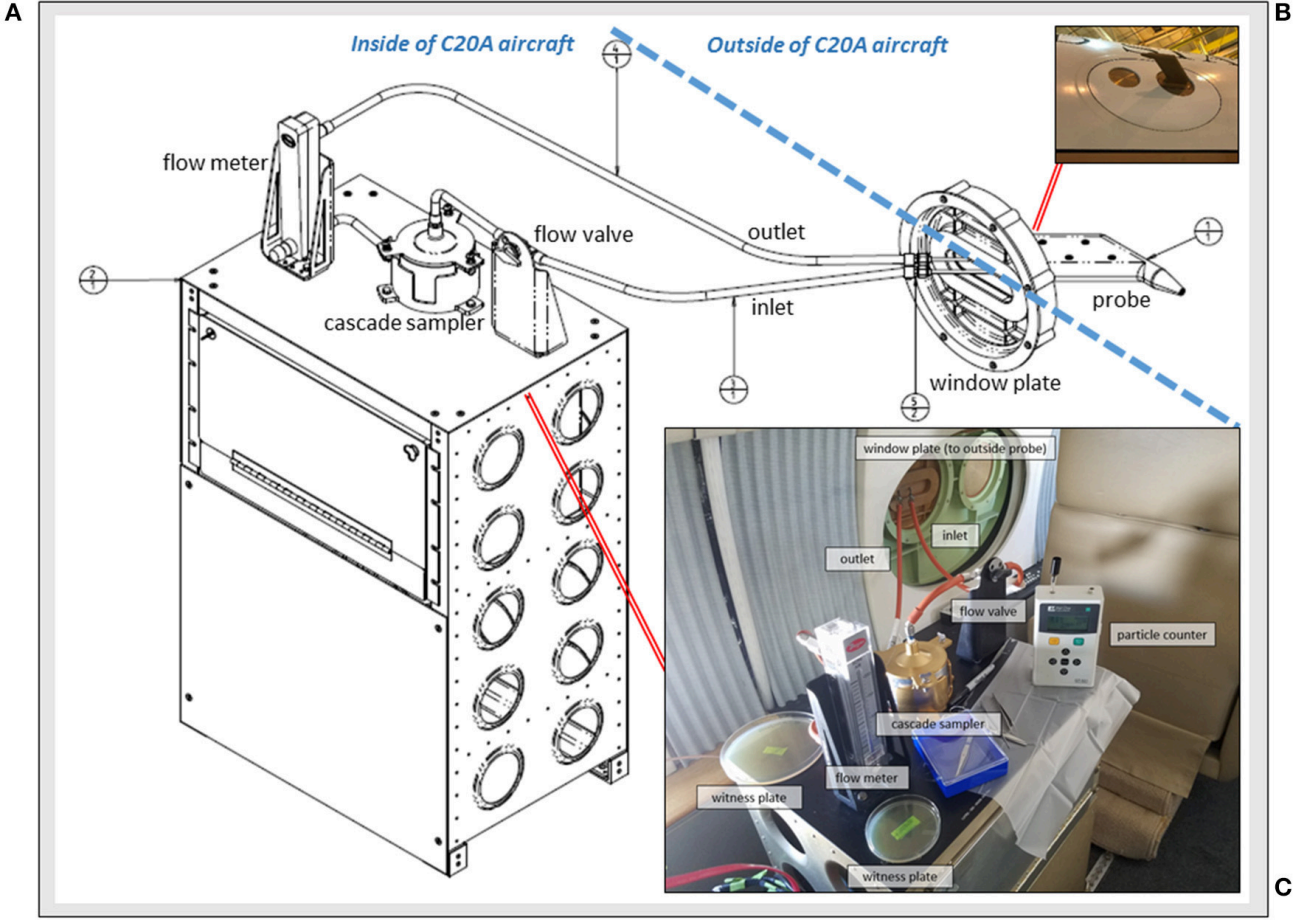
The Aircraft Bioaerosol Collector (ABC) system on the NASA C-20A aircraft. **(A)** Engineering diagram for components inside and outside of the aircraft; **(B)** Photograph of the window plate mounted probe for capturing free stream air; and **(C)** View from inside of the C-20A during in-flight operations with system elements labeled.

Accordingly, the primary aim of our study was to develop a low cost, reproducible window-mounted aircraft hardware system (Figure 1A) for microbiology collections in the upper troposphere and lower stratosphere. Our new system, the Aircraft Bioaerosol Collector (ABC), was designed to (1) function at extreme altitude and high aircraft velocities; (2) capture free stream atmospheric air samples at subzero temperatures; (3) regulate air flow (on/off) for collections at specific altitudes of interest; (4) use DNA-treated collection filters, replaceable in flight; (5) size separate bioaerosols; and (6) have components that could be easily installed, removed and periodically cleaned. After building and installing the ABC, we flew 4 test flights over the continental USA at altitudes up to 12.2 km demonstrating its operability. We sampled above and below the lower stratosphere to test the hypothesis that the tropopause serves as Earth's naturally occurring altitudinal biosphere boundary; specifically, we expected a significant drop in bioaerosol abundance and richness in the lower stratosphere (~12 km) compared to the troposphere (0.3 to 12 km). To evaluate our hypothesis we characterized airborne bacteria using molecular and culture-based methods, and we determined the atmospheric transport history of sampled air using meteorological models and satellite data.

## Materials and methods

### Aircraft description

NASA's C-20A Gulfstream III aircraft is available through the Airborne Science Program at Armstrong Flight Research Center (AFRC). The aircraft can reach altitudes of 13.7 km and cover a range of 6.3 km with an air speed up to 237 m·s^−1^. Flights can last 5–7 h and carry about 1,000 kg of useful scientific payload. During the course of our study, C-20A ride-along flight opportunities were provided by AFRC and the Uninhabited Aerial Vehicle Synthetic Aperture Radar (UAVSAR) team from NASA Jet Propulsion Laboratory (Koo et al., 2012). We flew 4 research flights across regions of the western USA: Eel River (Science Flight #955); California San Andreas Fault (CSAF) (Science Flight #956); Slumgullion (Science Flight #957); and LA Basin (Science Flight #958). Each flight path remained above the state of California (CA) except for the Slumgullion flight that also crossed over Arizona, Nevada, Utah, and Colorado.

### Aircraft bioaerosol collector (ABC)

Sampling free stream air (King, 1984) was an essential system design feature. The atmospheric probe (Figure 1B) was a miniaturized version of larger pitot-style inlets used on the NASA DC-8 aircraft for collecting atmospheric aerosols < 4 μm (Talbot et al., 1998; Dibb et al., 1999; Scheuer et al., 2003). STAR-CCM+ modeling (Siemens PLM Software, Plano, TX) and aerodynamic calculations were performed to determine a position for the probe that would pass through free stream air uninfluenced by C-20A engines or surfaces. We built a custom 29 cm (*l*) x 16 cm (*w*) x 3 cm (*h*) probe from aluminum (AL7050-T7452 per AMS 4050) with an inner diameter of 0.3 cm and clam shell construction that was secured with 6 screws (NAS11530-9), sealed using AMS-S-8802 sealant, and anodized (MIL-A-8625 type II). When mounted, the probe mass was ~1 kg and it was positioned 16 cm from the window plate, angled at 1.5 degrees below horizontal.

Depicted in Figure 1C, the probe-captured air traveled through hose lines (Part # 101001-3CR-0240; 101001-3CR-0065; 101001-3CR-0064; 101003-3CR-0360, Aviall Hose Shop, Van Nuys, CA) and into a sterilized 2-stage cascade sampler (Product # TE-10-860; Tisch Environmental, Cleves, OH) with successive aluminum stages fastened together by a butterfly cap and clamp system with silicone o-rings for air-tight sealing (039S70 and 044S70). The cascade sampler sat on a custom-built workbench containing all system components and supplies needed for flight operations. We modified the top portion of the sampler with quick release fasteners to allow for filter replacement in flight. Each sampler stage had 400 small round drilled orifices (1.18 mm on first stage; 0.25 mm on second stage). Gamma-irradiated (i.e., DNA-treated) gelatinous filter membranes (Part #16799-1001-02500, Sartorius, Bohemia, NY) sat underneath each aluminum stage for bioaerosol capture. Each filter was supported by a manufacturer-provided grid of polyethylene that was cut down to size and sterilized before use with a 90% isopropyl alcohol rinse (Sigma-Aldrich, St. Louis, MO). Plating the sterilized support grids on R2A (Difco, Sparks, MD) media did not yield any bacterial growth after 1 week of incubation at 25°C. Once atmospheric air traveled across the cascade sampler stages and gelatinous filter membranes, it then passed through a volumetric flow meter (Model RMC, Dwyer, Michigan City, IN). Flow rates were monitored during each flight and averaged 8.5 l·min^−1^ at the aircraft's cruise altitude of ~12 km. After passing the flow meter, air traveled into an exhaust line and back out the window port. To prevent air flow during take-off or landing, a ball valve (Part # 2F-B2LJ2-SSP-LD, Valin, San Jose, CA) was installed in-line and upstream of the cascade sampler.

### Experimental design and sample description

**Figure 2:**
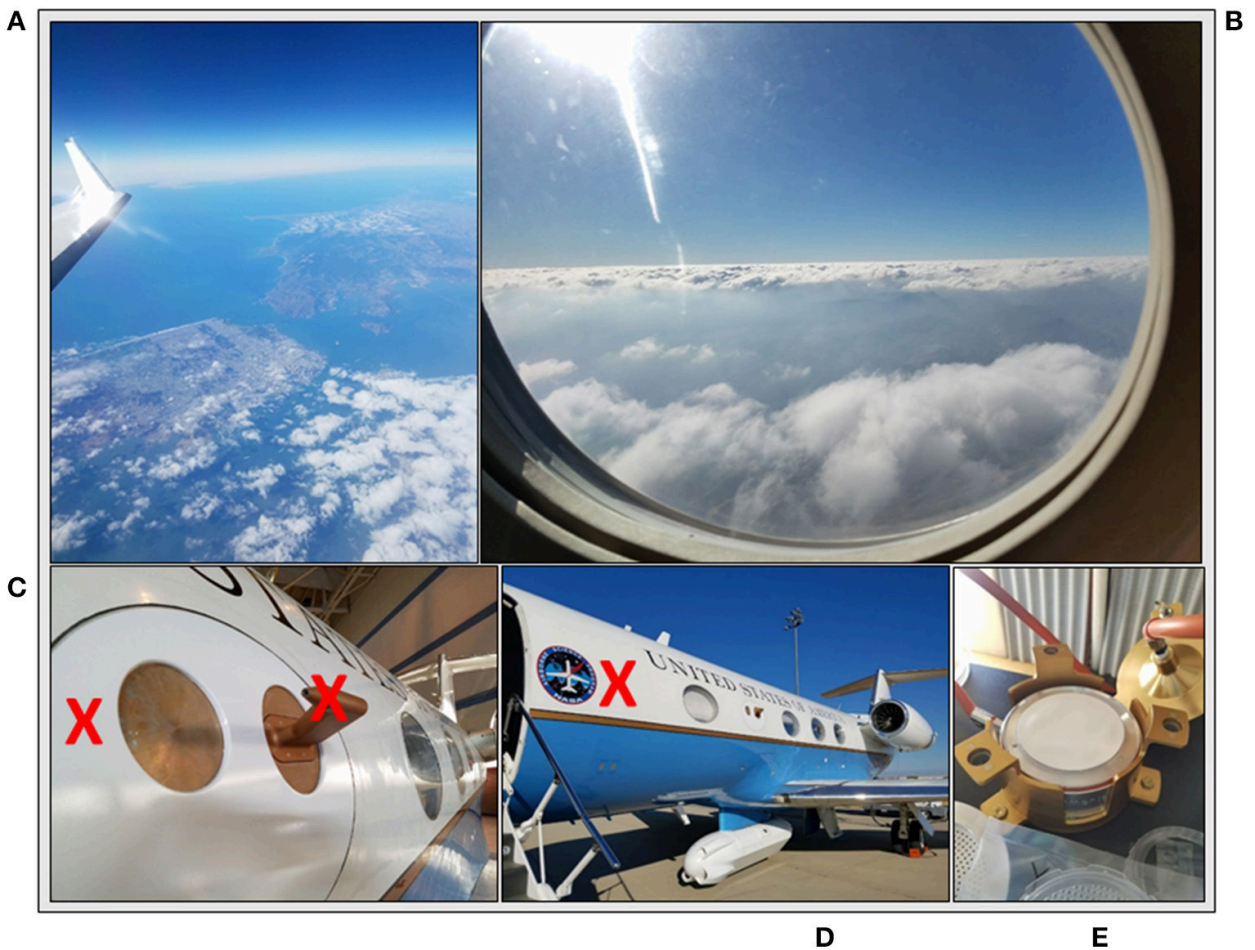
Experimental design overview. **(A)** Cruise samples at ~12 km; **(B)** Ascent/Descent samples ranging from 0.3 to 12 km; **(C)** Ground hardware samples (probe and window plate, *red X* indicating area swabbed); **(D)** Ground samples (side of aircraft, *red X* indicating area swabbed); and **(E)** Negative control sample where blank filter was loaded into cascade sampler.

Table 1 summarizes the collections in our study and Figure 2 shows photographs of the sample locations. Prior to flying, accessible ABC components and tools were sterilized by autoclaving or with isopropyl alcohol rinses. The window plate and probe inlet were assayed for contaminants using a pre-wetted sterile applicator (part # 25-8062WC, Puritan, Guilford, ME) before the C-20A departed the aircraft hangar; these samples were *Hardware* controls. Swabs were stored in 5 ml of sterile deionized water within a 15 ml Falcon tube and kept at 4°C until laboratory processing. Following the swab assay, the probe inlet was rinsed with isopropyl alcohol and sprayed dry with sterile air canisters. Before take-off and after landing, an exterior portion of the C-20A directly upstream of the probe was assayed using the swab method; these samples were *Ground* controls. The same area was sampled on consecutive days of flight operations to determine the bioburden of airborne microorganisms settling onto the C-20A during fueling, take-off and landing operations.

**Table 1:** Summary of samples.

	**Date (2017)**	**Flight**	**Type**	**Time (min)**	**CFUs**	**PCR Yield (ng·μl^−1^)**	**Notes**
1	30 Oct	Eel River	Ground	–	4	20.99	Ground, pre-flight, side of aircraft
2	30 Oct	Eel River	Ground	–	1	20.99	Ground, post-flight, side of aircraft
3	30 Oct	Eel River	Atmosphere	42	1	12.82	Ascent/Descent, in-flight, top stage of sampler
4	30 Oct	Eel River	Atmosphere	42	0	20.11	Ascent/Descent, in-flight, bottom stage of sampler
5	30 Oct	Eel River	Atmosphere	141	0	20.66	Cruise, in-flight, top stage of sampler
6	30 Oct	Eel River	Atmosphere	141	0	20.99	Cruise, in-flight, bottom stage of sampler
7	31 Oct	CSAF	Ground	–	7	20.99	Ground, pre-flight, side of aircraft
8	31 Oct	CSAF	Ground	–	7	14.22	Ground, post-flight, side of aircraft
9	31 Oct	CSAF	Atmosphere	41	0	3.43	Ascent/Descent, in-flight, top stage of sampler
10	31 Oct	CSAF	Atmosphere	41	1	0.01	Ascent/Descent, in-flight, bottom stage of sampler
11	31 Oct	CSAF	Atmosphere	250	0	0.28	Cruise, in-flight, top stage of sampler
12	31 Oct	CSAF	Atmosphere	250	0	0.86	Cruise, in-flight, bottom stage of sampler
13	1 Nov	Slumgullion	Ground	–	0	3.31	Ground, pre-flight, side of aircraft
14	1 Nov	Slumgullion	Ground	–	0	2.82	Ground, post-flight, side of aircraft
15	1 Nov	Slumgullion	Atmosphere	43	0	9.88	Ascent/Descent, in-flight, top stage of sampler
16	1 Nov	Slumgullion	Atmosphere	43	0	8.59	Ascent/Descent, in-flight, bottom stage of sampler
17	1 Nov	Slumgullion	Atmosphere	240	6	8.05	Cruise, in-flight, top stage of sampler
18	1 Nov	Slumgullion	Atmosphere	240	0	8.1	Cruise, in-flight, bottom stage of sampler
19	2 Nov	LA Basin	Ground	–	2	18.13	Ground, pre-flight, side of aircraft
20	2 Nov	LA Basin	Ground	–	0	19.62	Ground, post-flight, side of aircraft
21	2 Nov	LA Basin	Atmosphere	52	0	11.63	Ascent/Descent, in-flight, top stage of sampler
22	2 Nov	LA Basin	Atmosphere	52	0	20.4	Ascent/Descent, in-flight, bottom stage of sampler
23	2 Nov	LA Basin	Atmosphere	181	0	1.11	Cruise, in-flight, top stage of sampler
24	2 Nov	LA Basin	Atmosphere	181	0	11.75	Cruise, in-flight, bottom stage of sampler
25	2 Nov	LA Basin	Ground	–	0	10.13	Negative control, blank filter loaded into upper stage of sampler
26	2 Nov	LA Basin	Ground	–	0	8.24	Negative control, blank filter loaded into lower stage of sampler
27	30 Oct	Eel River	Ground	–	1	14.84	Hardware, pre-flight, probe sample
28	30 Oct	Eel River	Ground	–	0	20.99	Hardware, pre-flight, window plate sample
29	30 Oct	Eel River	Ground	–	0	19.24	Hardware, post-flight, probe sample
30	30 Oct	Eel River	Ground	–	2	11.8	Hardware, post-flight, window plate sample

Each flight day, the C-20A departed from and returned to AFRC (Palmdale, CA). Free stream atmospheric air collection began ~300 m above ground level after take-off by opening the ABC ball valve and flow meter. Upon reaching 10 km (~10 min into each flight), air flow for *Ascent* samples was stopped and new filters for *Cruise* samples were aseptically loaded into the cascade sampler for collections at the sustained altitude of ~12 km which lasted 141–250 min during the flight campaign. Two types of agarose witness plates (TSA and R2A, Remel, Fremont, CA) were installed on the workbench and exposed to ambient aircraft cabin air during flight operations to assess the amount of contamination inside the cabin air influencing filters during installation and removal procedures. A particle counter (Model GT-521, Met One Instruments, Inc., Grants Pass, OR) with 0.3 and 0.5 μm counting channels measured on average 13,400 particles and 300 particles, respectively, in the aircraft cabin air. Used filters were stored inside sterile Whirl-Paks (Part #B01297, Nasco, Modesto, CA) and kept inside an insulated cooler at 4°C. After the Cruise sample collection and prior to aircraft *Descent* below 10 km, the Ascent sample filters were returned into the cascade sampler, combining the Ascent & Descent samples for this study. The decision to pool *Ascent/Descent* samples (300–10 km) was made in order to focus our analysis on the potential difference between bacterial concentration and diversity in the troposphere vs. lower stratosphere. Air flow into the ABC was stopped again at ~300 m above ground level on runway approach for the C-20A at AFRC. Upon completion of each flight, cascade sampler components were cleaned with isopropyl alcohol; swabs and filters were kept at 4°C until transport back to NASA Ames (Moffett Field, CA).

### Microbiological methods

#### Sample concentration

Using sterile scissors and forceps, gelatinous filters were halved inside a Class II Type A Biosafety Cabinet (NU-540-600, Nuaire, Plymouth, MN). For each sample, one filter half was archived in a −80°C freezer (TSU-600A, Thermo Scientific, Asheville, NC) and the other half was dissolved in 40 ml of warm (37°C) molecular grade water (H2OMB0124, Millipore, Billerica, MA). Next, we used a concentrating pipette (CP Select, InnovaPrep, Drexel, MO) which passed the entire dissolved sample volume (40 ml) through a 0.1 μm flat membrane polyethersulfone membrane (part number CC08001), followed by elution with Tris buffer into a final output volume of 1 ml. Similarly, swabs contained in 15 ml tubes (wetted with 5 ml of sterile water) from Ground and Hardware control samples obtained from the window plate, probe, and side of the aircraft were concentrated in 1 ml of Tris. For each concentrated volume, 800 μl of was used for DNA extraction and subsequent 16S V4 sequencing; 100 μl was used for culture-based recovery assays; and 100 μl was archived at −80°C in the freezer.

#### Recovery of viable isolates and identification

Concentrated 100 μl aliquots were evenly spread onto R2A to encourage the recovery of viable heterotrophic bacteria. All samples were wrapped with Parafilm (American National Can, Chicago, IL) and placed in a dark incubator (SHKE6000, MaxQ 6000, Thermo Scientific, Manetta, OH) at 25°C for 2 weeks while monitoring for signs of growth. Table 1 summarizes the number of colony forming units (CFUs) for each sample. Individual colonies were sub-cultured on R2A until isolated and cryopreserved with 10% sterile glycerol (Amresco, Solon, OH) and nutrient broth (Difco, Sparks, MD) at −80°C. Deoxyribonucleic acid (DNA) extraction was performed on each isolate, followed by the polymerase chain reaction (PCR) amplification of 16S ribosomal ribonucleic acid (rRNA). A 466 bp 16S universal primer set from Nadkarni et al. (2002) [forward primer: 5′-TCCTACGGGAGGCAGCAGT-3′ (T_m_, 59.4°C); reverse primer: 5′-GGACTACCAGGGTATCTAATCCTGTT-3′ (T_m_, 58.1°C)] was used to generate bacterial amplicons. Sequence data from GENEWIZ (South Planfield, NJ) were then mapped to the most probable taxonomic affiliation of the bacteria using the Basic Local Alignment Search Tool (BLAST)^1^.

#### 16S V4 sequencing

DNA from each 800 μl concentrated sample aliquot was extracted using an AllPrep PowerViral DNA/RNA kit (product # 28000-50, Qiagen) then quantified by the Quant-iT PicoGreen dsDNA Assay Kit (Invitrogen, Life Technologies, Grand Island, NY). PCR amplification of the V4 hypervariable region of the bacterial 16S rDNA gene enriched samples prior to MiSeq (Illumina, San Diego, CA) sequencing. Post-amplification yields averaged 12.2 ng·μl^−1^ across samples with the upper limit of the reaction at 20.99 ng·μl^−1^ based on the ladders utilized. For library construction and normalization, we used two differently bar coded V4 fusion primers designed against the surrounding conserved regions tailed with sequences to incorporate Illumina adapters and indexing barcodes. Post amplification, each sample was quantified by fluorometric methods (Qubit or PicoGreen from Invitrogen, Life Technologies, Grand Island, NY) before sequencing. Every amplicon (containing 16S V4 enriched, amplified, barcoded samples) was loaded into a single MiSeq cartridge and flow cell for paired-end sequencing runs.

After sequencing, a bioinformatics analysis by Second Genome Inc. (South San Francisco, CA) filtered and trimmed all reads, followed by mapping to taxonomic databases for identifying bacterial composition. Detailed explanations for statistical methods, including quality control and assurance techniques, have been described elsewhere (Benjamini and Hochberg, 1995; Anderson, 2001; Oksanen et al., 2010; McDonald et al., 2012; Edgar, 2013; McMurdie and Holmes, 2013, 2014; Love et al., 2014). Briefly, a custom software package pre-processed, summarized, and normalized data followed by calculations of alpha diversity metrics (within sample diversity), beta diversity metrics (sample-to-sample similarity), ordination/clustering, sample classification, and significance testing. Representative Operation Taxonomic Unit (OTU) sequences were assigned using mothur's bayesian classifier, with clusters referenced at 99% alignment to the Greengenes^2^ database of 16S gene sequences. For additional information on reproducing the data pipeline we refer readers to Mohan et al. (2016), Alhasson et al. (2017), and Reveles et al. (2017). Access to raw sequencing data can be downloaded in the supporting files associated with this project archived at GeneLab^3^ using accession GLDS-170. To account for possible contaminants associated with the experimental design, filtered taxonomic tables were generated after removing a subset of OTUs identified from extraction-negative and no-template PCR control samples. Both raw and filtered OTU tables are available in the GLDS-170 project folder.

### Environmental data and atmospheric modeling

We collected GPS position, altitude, wind speed, aircraft speed, and air temperature data from the C-20A during flight operations. To understand the transport history of the air masses sampled, we calculated 2-day kinematic back-trajectories over the flight paths using the Hybrid Single-Particle Lagrangian Integrated Trajectory (HYSPLIT) model (Stein et al., 2015) which uses global meteorological data from the Global Data Assimilation System archive. Trajectories were run at 3 heights (300, 5,000, and 12,000 m above ground level). In order to gain 1 km-scale resolution of the vertical distribution of aerosols and components (e.g., SO_4_, organic carbon, black carbon, dust, sea salt), we retrieved satellite data from MODIS and produced aerosol optical depth (AOD) maps at 470 nm based on the MAIAC retrieval algorithm (Lyapustin et al., 2011). These resulted in a 3-D model of aerosol distribution along flight lines (matching latitude, longitude, and altitude) using MERRA-2 methods (Gelaro et al., 2017).

## Results

### Flight data and atmospheric modeling

**Figure 3:**
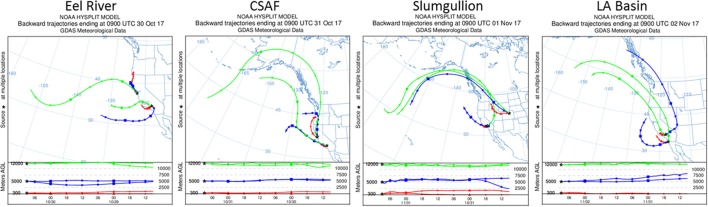
HYSPLIT kinematic back trajectories model air transport history for flights 30 Oct to 2 Nov 2017.

**Figure 4:**
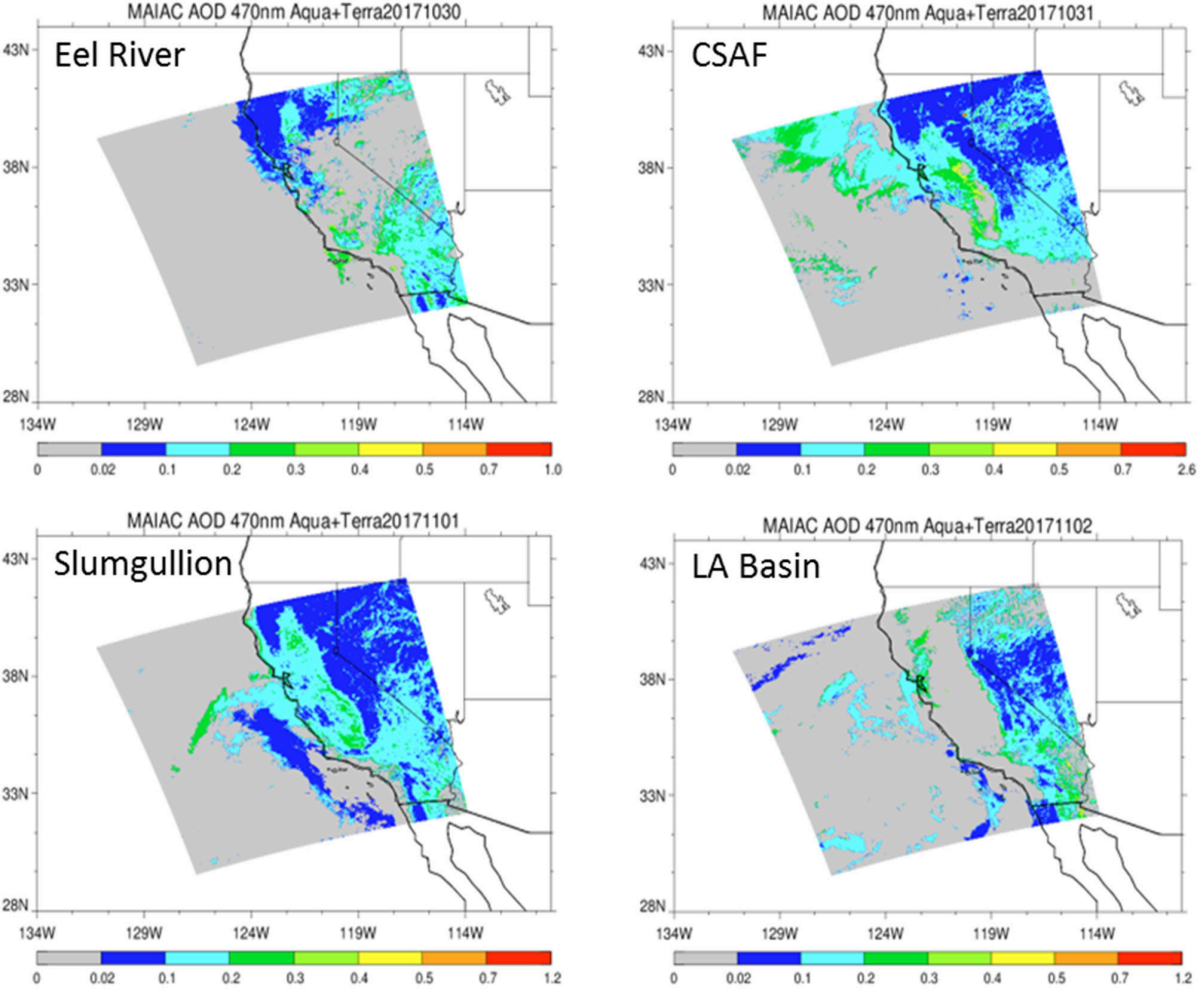
Aerosol Optical Depth (AOD) measurements at 470 nm based on the MAIAC retrieval algorithm derived from combined satellite datasets (MODIS/Terra and MODIS/Aqua) shows vertically integrated aerosols over relevant flight lines.

**Figure 5:**
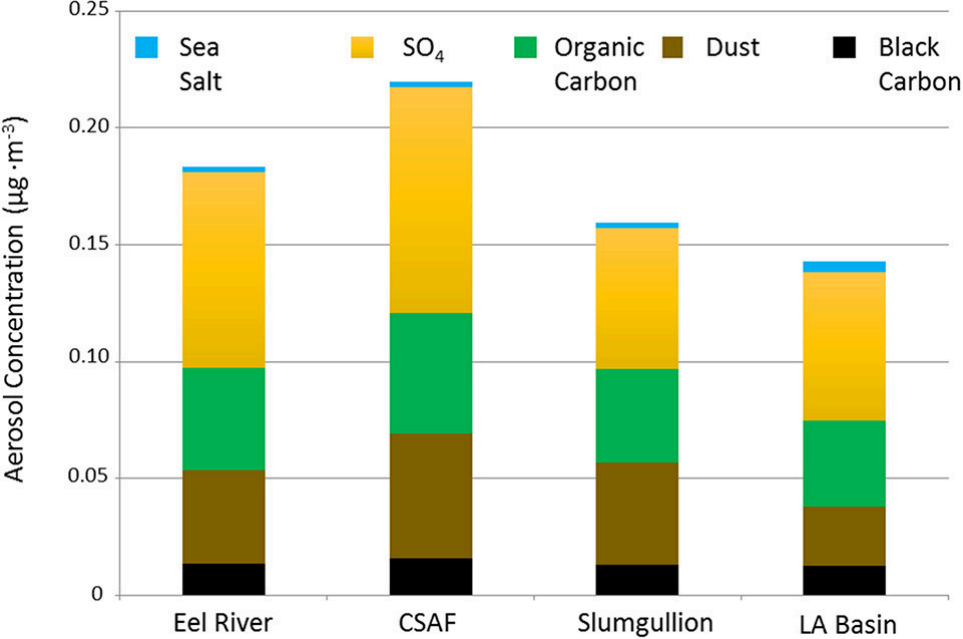
MERRA-2 data summarizing SO_4_, organic carbon, dust, black carbon, and sea salt aerosol concentrations averaged across the cruise altitude of ~12 km for each flight.

**Figure 6:**
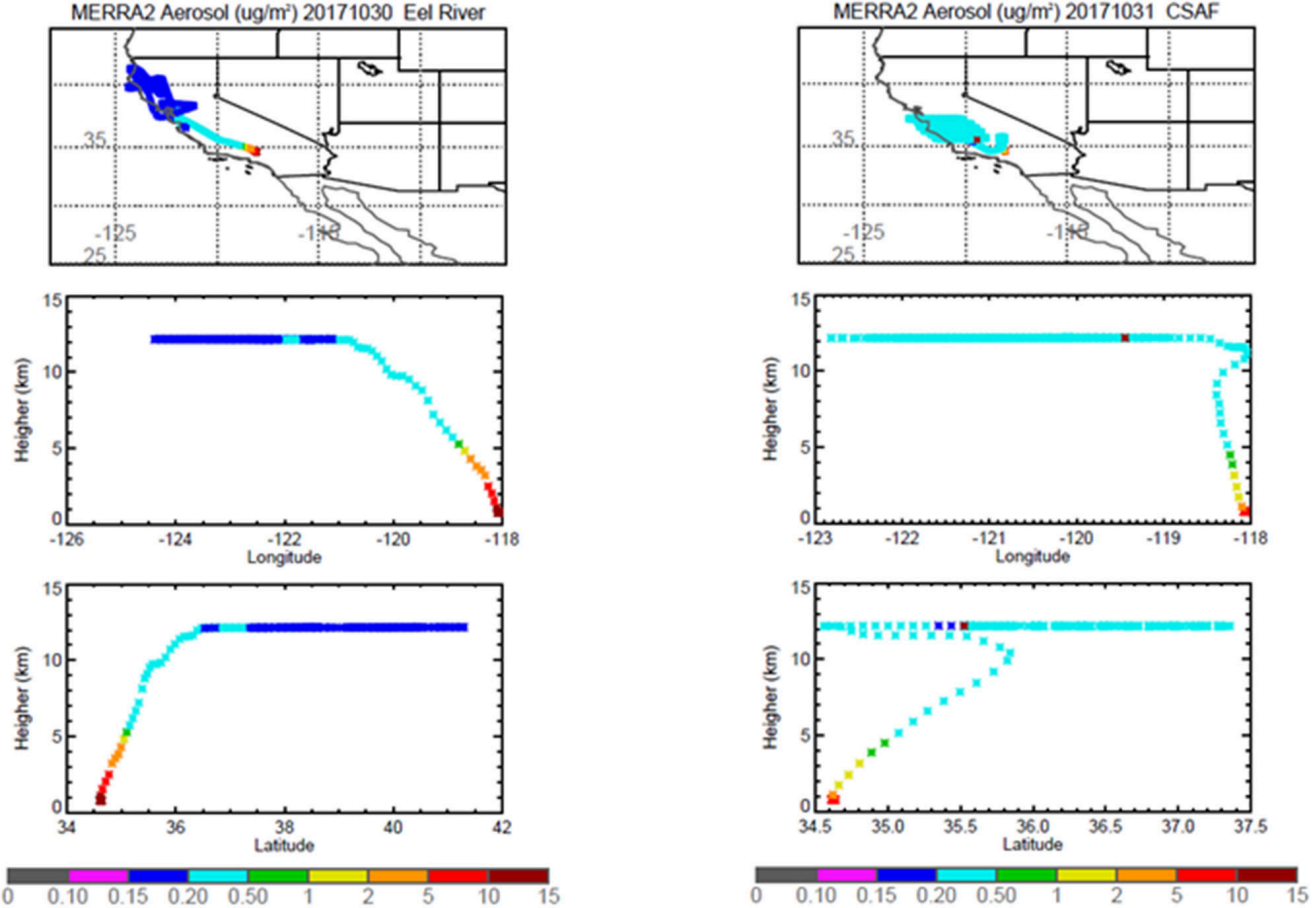
MERRA-2 results depicting bulk aerosol concentration (μg·m^−3^) for Eel River **(Left)** and CSAF **(Right)** flights; *top* panel shows horizontal cross section and *bottom* panel shows vertical cross section.

**Figure 7:**
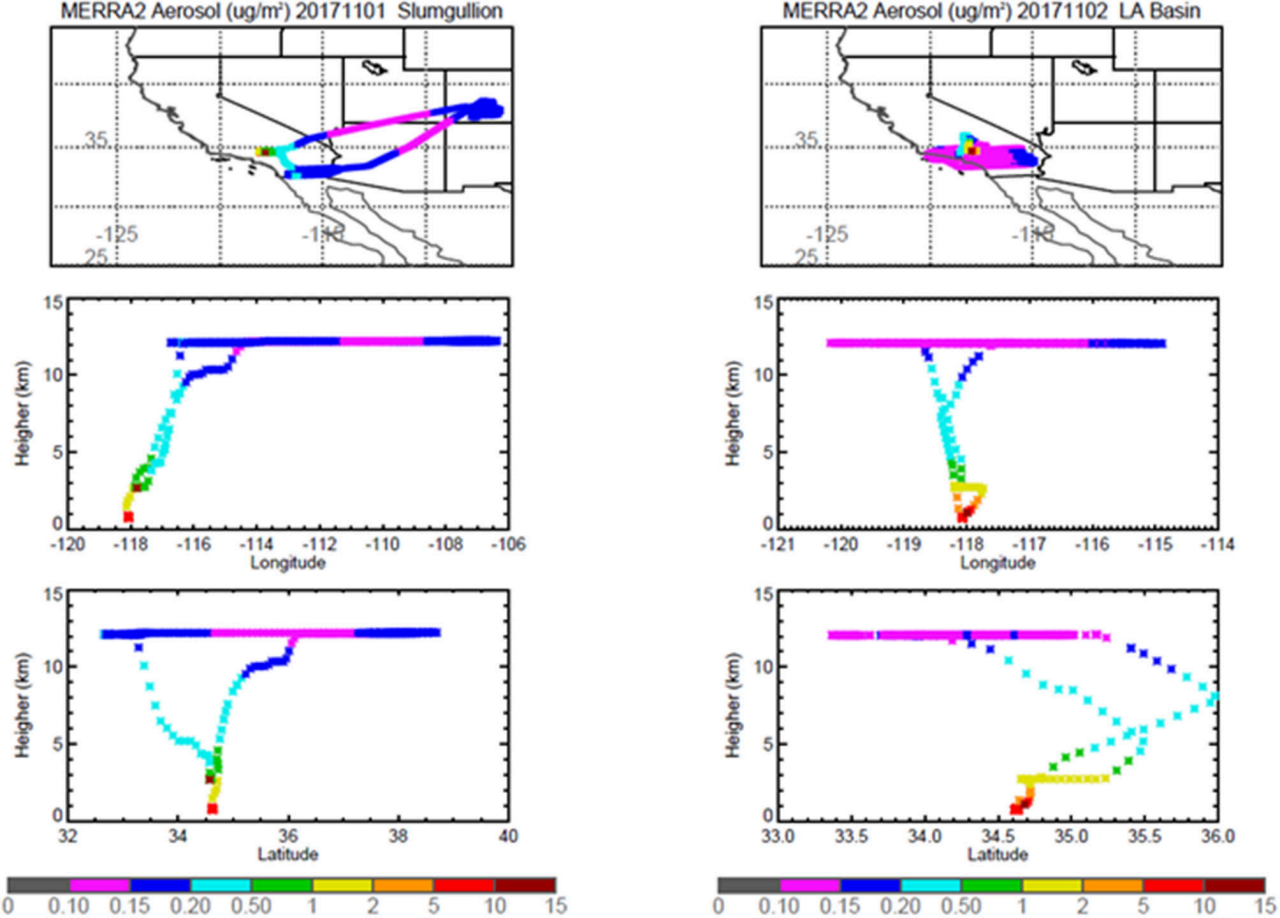
MERRA-2 results depicting bulk aerosol concentration (μg·m^−3^) for Slumgullion **(Left)** and LA Basin **(Right)** flights; *top* panel shows horizontal cross section and *bottom* panel shows vertical cross section.

Pertinent flight data are summarized in Table 2. The modeled air mass back trajectories and aerosol distribution suggest our flights passed through air with similar transport histories and bulk aerosol composition. Figure 3 depicts the HYSPLIT model based on GDAS meteorological data at heights and positions within the range of C-20A flight lines. Generally, the 48-h back trajectories resemble each other across the modeled heights, showing air masses traveling eastward off the Pacific Ocean into the US. Mostly uniform aerosol mass loading was observed across the flight lines (Figure 4) with Aerosol Optical Depth (AOD) measurements at 470 nm using the MAIAC retrieval algorithm derived from combined satellite datasets (MODIS/Terra and MODIS/Aqua). Satellite observations of modeled aerosol concentrations at ~12 km were higher during the Eel River (30 Oct 2017) and CSAF (31 Oct 2017) flights and lower on Slumgullion (1 Nov 2017) and LA Basin (2 Nov 2017) flights, consisting primarily of SO_4_, organic carbon, dust, black carbon, and sea salt (Figure 5). Satellite data also showed smoke influence near ground levels along the CSAF flight track. MERRA-2 aerosol simulations (Figures 6, 7) were produced using C-20A flight positions (latitude, longitude, and altitude) and were consistent with the MAIAC derived AOD distribution. MERRA-2 results show aerosol concentrations decreasing sharply with altitude (i.e., highest concentrations were in the lower atmosphere at Ascent/Descent). The concentration of aerosols was low (<0.5 μg·m^−3^) most of the flight time at Cruise altitudes of ~12 km. However, one exception at 12 km was the CSAF flight (31 Oct 2017) when the aerosol concentration briefly reached up to 10 μg·m^−3^.

**Table 2:** Environmental and positional data averaged across flights.

**Flight**	**Time (UTC)**	**Air Temp (^°^C)**	**Altitude (km)**	**Air Speed (m·s^−1^)**	**Wind Speed (m·s^−1^)**	**Wind Direction (^°^)**
Eel River	17:16 to 21:07	−26.7	12.15	231.3	17.3	308.9
CSAF	16:48 to 22:10	−28.9	12.17	225.4	13.2	127.2
Slumgullion	16:14 to 21:18	−32.4	12.17	226.0	16.6	287.1
LA Basin	17:34 to 23:09	−27.6	12.06	226.3	22.8	236.9

### Culture-based results

Most of the culturable bacteria collected in the study came from (Ground) swabs of the exterior portion of the C-20A before and after flights. Bacterial isolates included numerous *Bacillus carboniphilus, B. crescens, B. fumarioli, B. megaterium, B. aryabhattai, B. humi*, and *B. timonesis*, as well as *Gordonia paraffinivorans, Bhargavaea ginseng*, and *Streptomyces* sp. *Bacillus timonesis* was also obtained from a post-flight swab of the window plate where the ABC probe was mounted. Several bacterial isolates were recovered from in-flight collections, including *Bacillus* sp., *Micrococcus* sp., *Arthrobacter* sp., and *Staphylococcus* sp. from Cruise samples and *Brachybacterium* sp. from Ascent/Descent samples. However, none of the flight isolates were identifiable to the species level with Sanger sequencing of the 16S rRNA gene because of the relatively short region (~466 bp) covered by the assay.

Witness plates were used during each flight to monitor bacteria from C-20A cabin air settling onto the surface of the workbench. Although the ABC cascade sampler housed DNA-treated filters were sealed off from the cabin air during collection periods, filter change-out procedures exposed samples to cabin air for several minutes. Blank filters loaded did not yield any CFUs, however witness plates exposed to cabin air during the entire duration of flights (~250 min on average) resulted in a substantial amount of culture-based growth. The mean number of bacterial and fungal CFUs collected on witness plates was 0.663 CFU·cm^−2^.

### Culture-independent results: 16S V4 sequencing

Altogether, 2,557 OTUs were observed from 3,911,495 sequences. Of the OTUs, 98.86% of sequences were classified at the family level; 95.11% at genus; 2.837% at species; and 2.38% at strain. Prevalence filtering was applied to remove any spurious OTUs that were observed in less than 5% of the samples. After removal of the spurious OTUs, the number of total OTUs was reduced from 2,557 to 1,181 and the number of total sequences dropped from 3,911,495 to 3,886,523. The number of reads in the filtered library for Ascent/Descent group ranged from 2,470 to 160,341 (*N* = 97,689); Cruise group ranged from 12,693 to 707,558 (*N* = 188,541); Ground group ranged from 98,459 to 234,313 (*N* = 136,160); and Hardware group ranged from 108,177 to 147,995 (*N* = 133,094). After filtering, the number of unique categories at each taxonomic rank was 879 species, 609 genera, and 268 families.

Table 3 summarizes the eight most abundant phyla detected in the study (Firmicutes, Proteobacteria, Actinobacteria, Bacteroidetes, Cyanobacteria, an Unclassified phylum, Euryarchaeota, and Fusobacteria). Firmicutes and Proteobacteria, and to a lesser extent Actinobacteria, represent the majority of samples. Hardware samples had a slightly higher proportion of Proteobacteria and a lower abundance of Firmicutes compared to Ascent/Decent, Cruise, and Ground samples. The seven most abundant classes across all categories were Bacilli, Gammaproteobacteria, Betaproteobacteria, Clostridia, Actinobacteria, Erysipelotrichi, and Alphaproteobacteria. The eight most abundant families detected from all samples were *Staphylococcaceae, Moraxellaceae, Oxalobacteraceae, Lachnospiraceae, Ruminococcaceae, Comamonadaceae, Geodermatophilaceae*, and *Burkholderiaceae*.

**Table 3:** Mean and standard deviation (sd) values for the percent relative abundances of selected taxa at the Phylum level.

**Phylum**	**Ascent/Descent**	**Cruise**	**Ground**	**Hardware**
Firmicutes	80.6 (5.99)	78.1 (13)	76.1 (10.9)	65.1 (12.6)
Proteobacteria	16.7 (6.77)	20.1 (12.5)	19 (7.45)	29.7 (10.2)
Actinobacteria	0.887 (0.292)	0.798 (0.391)	3.67 (3.05)	3.92 (2.49)
Bacteroidetes	1.27 (1.5)	0.452 (0.133)	0.542 (0.169)	0.574 (0.0795)
Cyanobacteria	0.176 (0.164)	0.189 (0.202)	0.231 (0.258)	0.18 (0.165)
Unclassified	0.0751 (0.0931)	0.0691 (0.054)	0.0986 (0.0955)	0.0639 (0.0288)
Euryarchaeota	0.0757 (0.0837)	0.0865 (0.104)	0.0671 (0.0597)	0.0358 (0.0201)
Fusobacteria	0.111 (0.237)	0.0493 (0.0875)	0.0322 (0.0202)	0.0431 (0.0185)
Others	0.156 (0.139)	0.106 (0.0969)	0.308 (0.249)	0.394 (0.264)

**Figure 8:**
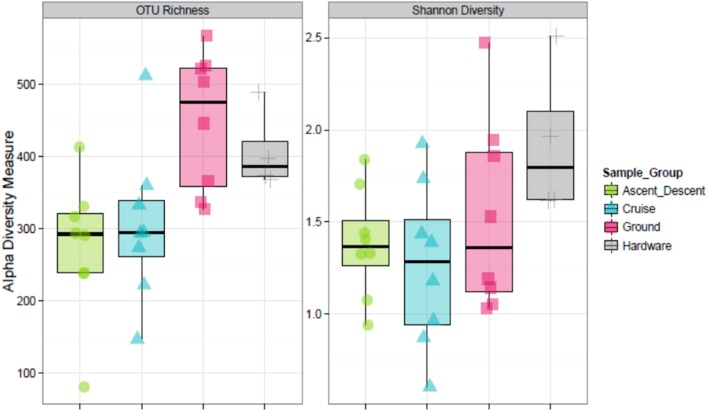
Alpha diversity estimates. **(Left)** OTU richness across sample groups. **(Right)** Shannon Diversity Index based on richness and evenness of OTUs within a sample.

Figure 8 depicts the richness of samples by showing the number of unique OTUs and Shannon diversity indices across each category based on abundance data. Means for OTU richness were higher for Ground (449; sd = 94.5) and Hardware (407; sd = 56) samples than flight samples from Ascent/Descent (276; sd = 96.3) and Cruise (305; sd = 107) altitudes. Table 4 summarizes the significant alpha diversity differences between groups using an unpaired Kruskal-Wallis test. There were significant differences between flight groups (Ascent/Descent and Cruise) compared to Ground and Hardware groups. Similar to the OTU richness results, Shannon diversity indices were also higher for Ground (1.53; sd = 0.523) and Hardware (1.93; sd = 0.421) samples compared to flight samples from Ascent/Descent (1.38; sd = 0.296) and Cruise (1.26; sd = 0.446).

**Table 4:** Alpha diversity differences across groups using an unpaired Kruskal-Wallis test for significance.

**Comparison**	***Z*-value**	***p*-value**
Ascent/Descent vs. Cruise	−0.043	0.6704
Ascent/Descent vs. Ground	−3.05	0.0023
Cruise vs. Ground	−2.63	0.0086
Ascent/Descent vs. Hardware	−2.22	0.0263
Cruise vs. Hardware	−1.87	0.061
Ground vs. Hardware	−0.027	0.7849

**Figure 9:**
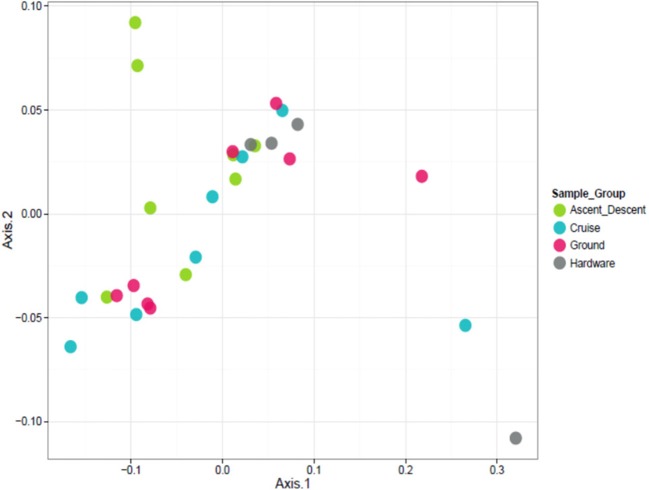
Weighted ordination based on relative abundance showed no clustering by sample group. The first two ordination axes accounted for 77.5% of sample variation.

**Figure 10:**
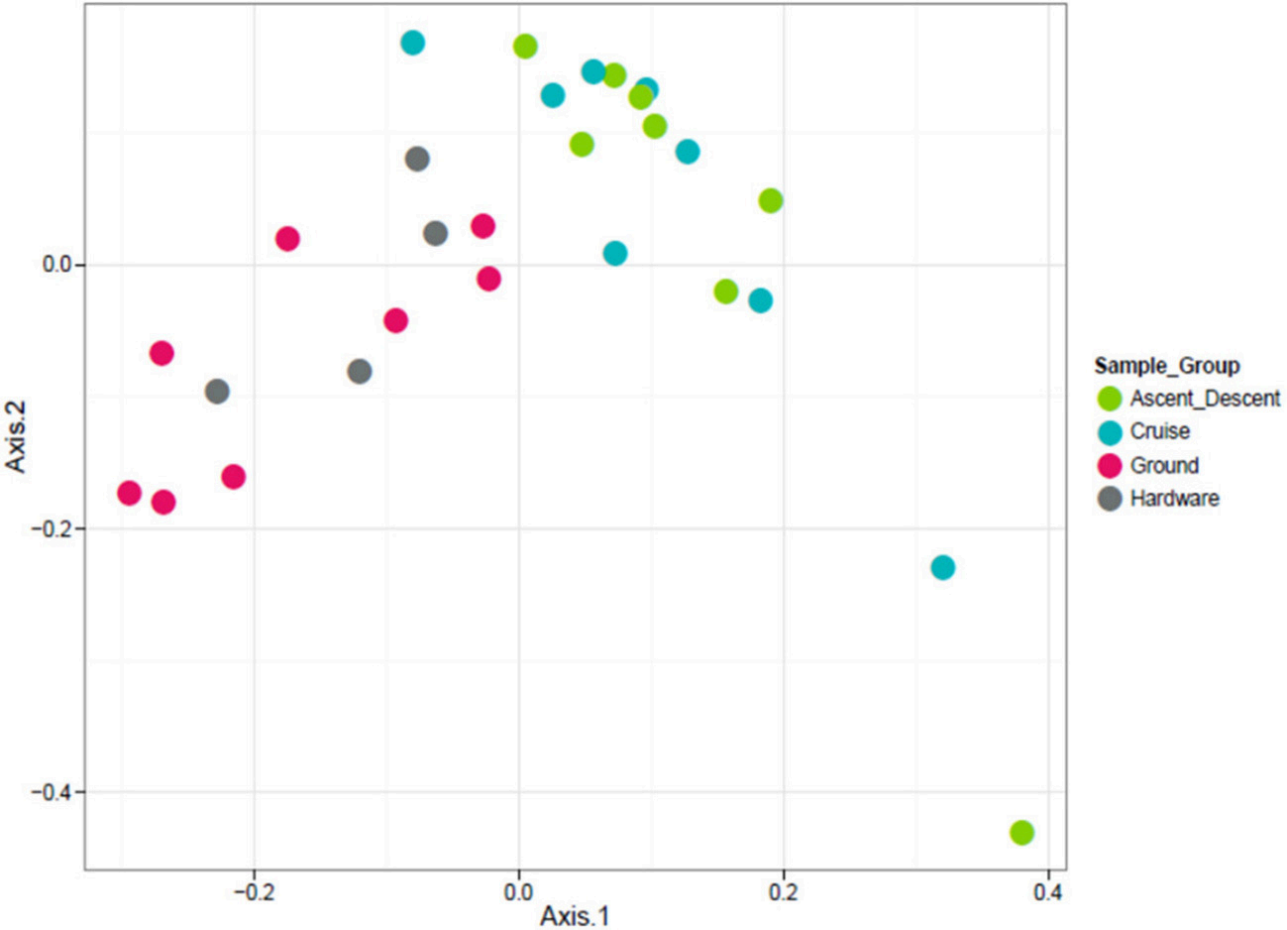
Unweighted ordination based on presence/absence showed non-significant clustering in sample groups. Flight samples (Cruise and Ascent/Descent) separated from the Ground and Hardware samples. The first two ordination axes accounted for 23.2% of sample variation.

To visualize the relationship between sample groups (Cruise, Ascent/Descent, Ground, Hardware) and ascertain sample-to-sample dissimilarity based on whole microbiome abundance profiles, we used a Principal Coordinate Analysis (PCoA). While the weighted ordination in Figure 9 (based on relative abundance) did not show clustering or any significant differences by sample group with a PERMANOVA analysis using Bray-Curtis dissimilarity (*p*-value = 0.081), an unweighted ordination in Figure 10 (based on presence or absence) depicted non-significant but noticeable differences across groups using Jaccard distance (*p*-value = 0.097). Specifically, the Ground and Hardware samples clustered separately from the Ascent/Descent and Cruise samples. One possible explanation for the perceived separation could be the relative enrichment in OTUs from phylum Actinobacteria in Ground samples, compared to the enrichment in OTUs from phylum Firmicutes in Ascent/Descent and Cruise samples. Ten strains identified as significantly enriched (adjusted *p*-value < 0.05) in Ground samples compared to flight samples potentially contributed to the separation between groups: *Blastococcus* sp. BC412; *Georgenia* sp. JC82; *Modestobacter multiseptatus*; *Modestobacter versicolor*; *Modestobacter marinus*; *Clostridium sordellii*; *Ornithinimicrobium kibberense*; *Yaniella* sp. G5; *Nocardioides* sp. MSL 22; and *Blastococcus jejuensis*.

**Figure 11:**
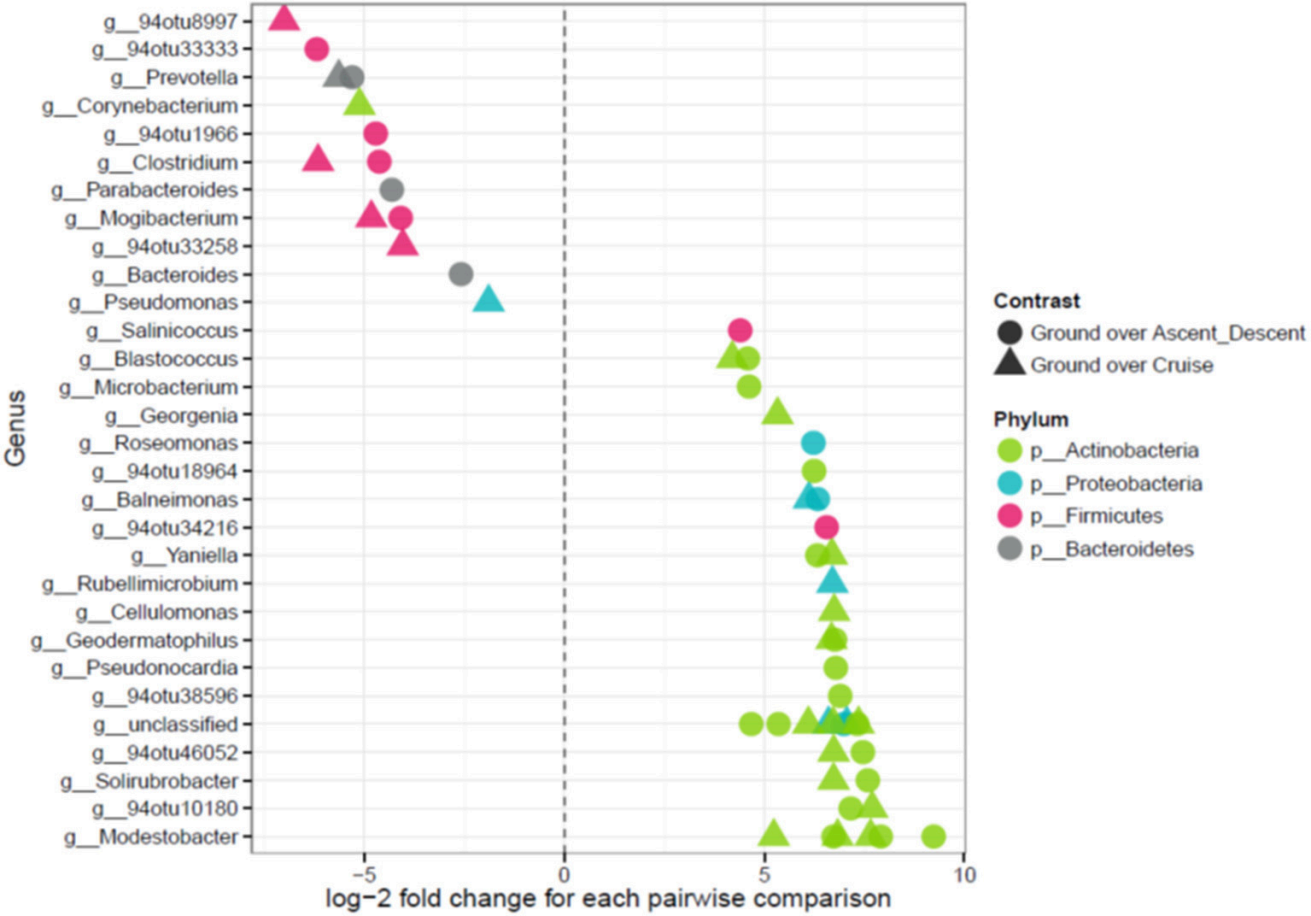
Points on the *left* depict OTUs in Ascent/Descent and Cruise samples that were more abundant compared to Ground samples; points on the *right* depict OTUs enriched in Ground samples. Features were considered significant if FDR-corrected *p*-values were < 0.05 and the absolute value of the log-2 fold change was ≥ 1.

Because the ordination data showed Ascent/Descent samples and Cruise samples were more similar to each other than different, Figure 11 was generated to highlight which OTUs stood out from Ground samples. A total of 28 OTUs were found to be differentially abundant between Ground samples and Ascent/Descent samples. Notably, OTUs from the families *Lachnospiraceae* and *Erysipelotrichaceae* and the genera *Clostridium, Mogibacterium, Bacteroides, Prevotella*, and *Parabacteroides* were more abundant in the Ascent/Descent samples than the Ground samples. When comparing differentially abundant OTUs in Ground samples against Cruise samples, a total of 25 OTUs were identified. The taxa more abundant in Cruise samples included OTUs from the families *Erysipelotrichaceae* and *Ruminococcaceae* and the following genera: *Clostridium, Mogibacterium, Corynebacterium*, and *Prevotella*. *Pseudomonas stutzeri* was the only OTU identified at the species level that was significantly more abundant in Cruise samples than Ground samples.

**Figure 12:**
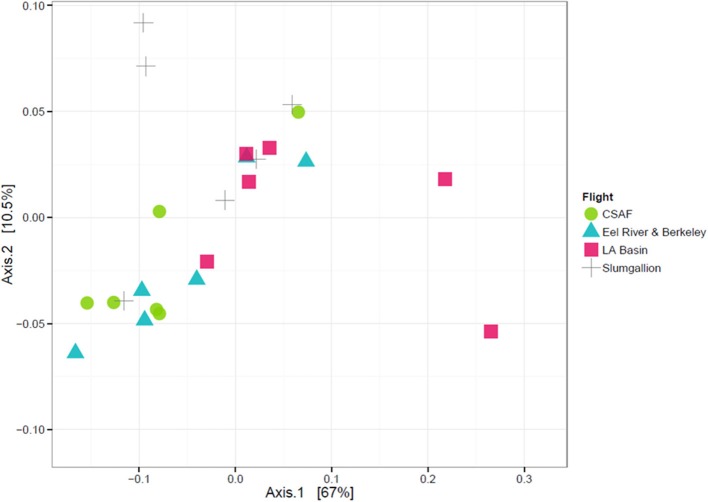
Weighted ordination plot based on OTU abundance showing that samples did not cluster by flight. The first two ordination axes accounted for 77.5% of sample variation.

**Figure 13:**
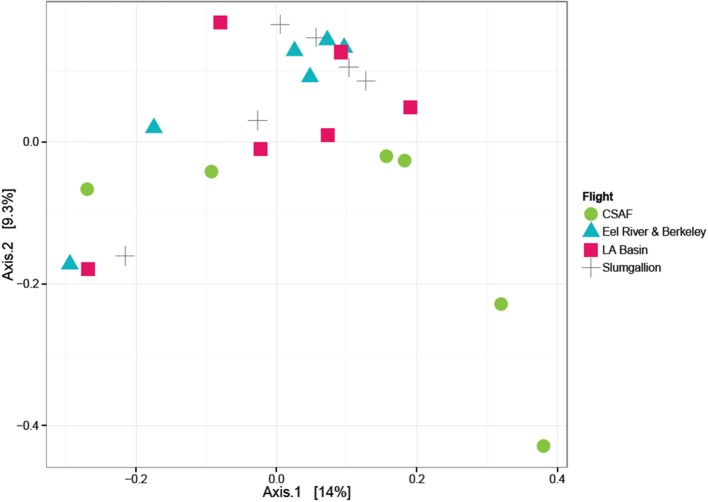
Unweighted ordination plot based on OTU presence/absence showing that samples did not cluster by flight. The first two ordination axes accounted for 23.2% of sample variation.

In order to determine if the differing daily flight paths contributed to microbiome diversity, we performed a PERMANOVA analysis for testing significant differences between each set of flight samples. Despite significant differences based on PERMANOVA (*p*-value = 0.013), a weighted ordination plot in Figure 12 using abundance data showed that flight samples did not cluster according to flight location. Airborne bacteria collected over Eel River, CSAF, Slumgullion, and LA Basin were more similar than different. An unweighted ordination shown in Figure 13 using presence/absence data also showed no clear clustering by flight.

**Figure 14:**
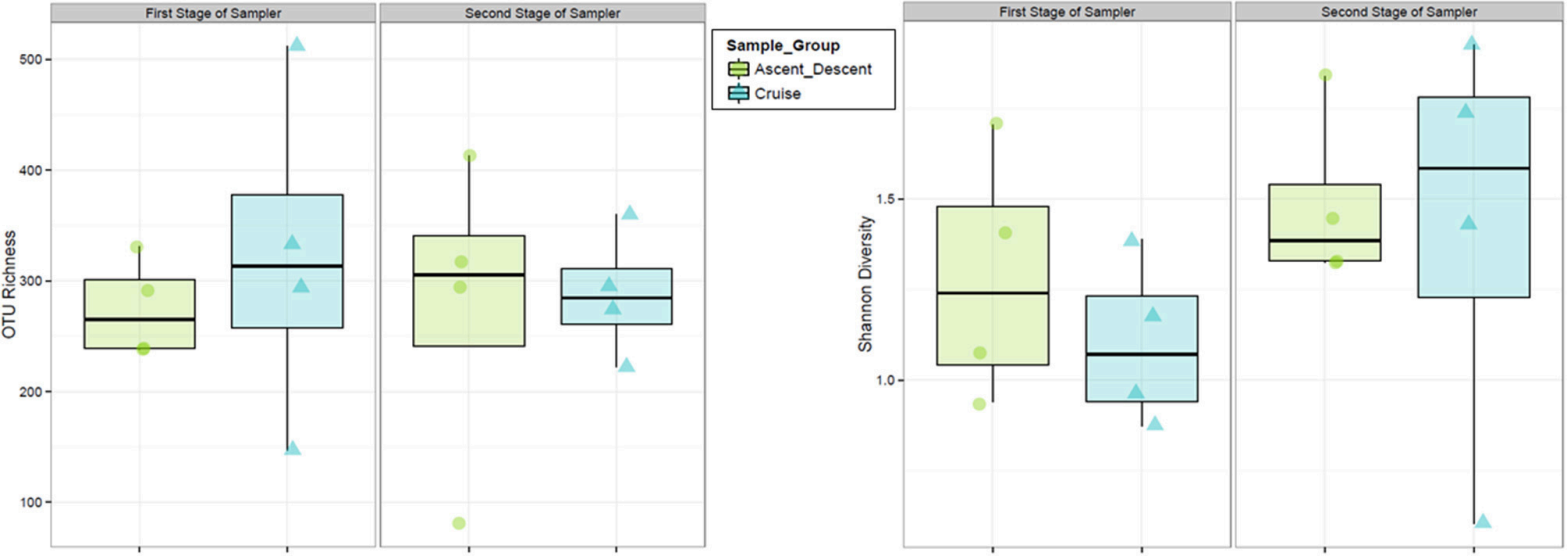
Relative abundance and diversity of flight samples (Ascent/Descent and Cruise) collected on the two internal stages of the ABC cascade sampler.

Finally, we analyzed samples on each of the two cascade sampler stages to ascertain if bacteria collected in the ABC were size sorted. The first stage of the sampler had 1.18 mm drilled orifices, the second stage had 0.25 mm drilled orifices. According to manufacturer specifications^4^, at ground-normal STP conditions using a flow rate of 28.3 L·min^−1^, the first stage would select for particles 5.8 to 9 μm and the second stage 0.7 to 1.1 μm. Figure 14 summarizes the OTU richness and Shannon Diversity between the two sampler stages. For samples collected on the first stage across all flights, the mean OTU richness was 275 (sd = 44.9) and 322 (sd = 150) for Ascent/Descent and Cruise groups, respectively. For samples collected on the second stage across all flights, the mean OTU richness was 276 (sd = 140) and 288 (sd = 57.1) for Ascent/Descent and Cruise groups, respectively. Without additional species-level identifications and microscopy data, we could not determine the size of bacterial cells, spores, and fragments contributing to OTUs measured.

## Discussion

Our results showed no clear differences in the richness or diversity of airborne bacteria collected at lower altitudes 0.3 to 12 km (Ascent/Descent samples) compared to higher altitudes ~12.1 km (Cruise samples). Since the bacteria from these regions were more similar than different, our initial hypothesis of the tropopause serving as a naturally occurring altitude boundary for bioaerosols was not supported. Instead, our data suggested that bacteria in the atmosphere up to 12 km were homogenously distributed. However, we had no way of testing whether a subset of bioaerosols from lower altitudes (i.e., Ascent/Descent samples) remained in the ABC inlet lines prior to the initiation of higher altitude collections. Such “carry-over” would be an alternative explanation for the even distribution of OTUs measured across the troposphere and lower stratosphere. While our new ABC system showed its ability to collect bioaerosols at extreme altitudes, the influence of contaminants from hardware remained apparent—thus, inherent limitations of our dataset and areas for improvements will be discussed later.

Although in relatively low abundance, the diversity of bacteria in the atmosphere seems to mirror taxa found in numerous terrestrial and aquatic ecosystems. The phyla most abundant in our study—Firmicutes, Proteobacteria, Actinobacteria, Bacteroidetes, Cyanobacteria, Euryarchaeota and Fusobacteria—are also reported widely across aerobiology literature, as reviewed by Amato et al. (2017a). Firmicutes (gram positive), Proteobacteria (gram negative), and to a lesser extent Actinobacteria (gram positive), represented the majority of OTUs and culture-based isolates detected in our survey. By far, the most common phyla was Firmicutes, with Bacilli and Clostridia classes in significant abundance, followed distantly by Erysipelotrichi. Firmicutes were also the predominant phyla in another upper atmosphere microbiology study conducted at an alpine observatory in central Oregon collecting transpacific bioaerosols (Smith et al., 2013). The relative abundance of *Bacillus* sp. and *Staphylococcus* sp. was expected since desiccated soils and desert dust transport embedded microorganisms into the atmosphere (Griffin et al., 2017). In addition to *Staphylococcus* sp., many of the atmosphere sampled OTUs from the *Lachnospiraceae* and *Ruminococcaceae* families are associated with fecal matter from agriculture fields, livestock feedlots, and human wastewater. Recent work by Bowers et al. (2013) also measured these taxa in high abundance throughout the atmospheric boundary layer. Many of the phyla from our flight samples, including Alpha- Beta- and Gammaproteobacteria, Firmicutes, and Bacteroidetes, are commonly found in air samples adjacent to coastal regions (Urbano et al., 2011). With most of our flights occurring over the state of California, the prevailing wind direction (traveling eastward) might have carried marine bioaerosols from the Pacific Ocean. Indeed, sea salt was present in the aerosol composition modeled at ~12 km for all flight days. *Moraxellaceae* was the most abundant family from Gammaproteobacteria measured in our study samples; *Oxalobacteraceae, Comamonadaceae*, and *Burkholderiaceae* were abundant families in the Betaproteobacteria class. While their overall abundance was very low in our study (< 1%), the detection of Cyanobacteria (phototrophic), Euryarchaeota (methanogenic) and Fusobacteria (potentially pathogenic), remains noteworthy considering the wide range of phenotypes and environmental impacts of these phyla.

*Clostridium, Mogibacterium, Bacteroides, Prevotella, Parabacteroides*, and *Corynebacterium* were significantly more abundant in flight samples (Ascent/Descent and Cruise) than in ground samples. The genus *Clostridium* is known for endospore forming bacteria, an adaptation known to enhance the resistance to biocidal factors in the atmosphere such as ultraviolet light, desiccation, and freeze/thaw cycles. Another endospore-forming genus measured in our culture-based isolates was *Bacillus*. However, interestingly, it was not among the most abundant genera measured with 16S V4 sequencing perhaps due to the inefficiency of lysing bacterial endospores with commercial DNA extraction kits. Besides *Clostridium*, genera identified as more abundant in flight samples (based on culture-independent sequencing) were non-spore forming OTUs from *Mogibacterium, Bacteroides, Prevotella, Parabacteroides*, and *Corynebacterium*. *Mogibacterium* and *Corynebacterium* are Gram-positive bacteria while *Bacteroides, Prevotella*, and *Parabacteroides* are Gram-negative bacteria; these genera are widely distributed in nature and commonly found in animal gut and oral microbiomes. Based on the prevalence of these genera, wastewater treatment facilities and agriculture producing aerosolized fecal matter could be possible sources of bacteria captured at high altitudes in this study; but establishing connections to specific emission sources was not achievable within our experimental framework.

Altogether, our study's detection of airborne bacteria in the lower stratosphere supports other reported evidence of microorganisms present at extreme altitudes (Griffin, 2004; Smith et al., 2009). In addition to *Bacillus* sp., other viable isolates recovered from lower stratosphere samples included *Micrococcus* sp., *Arthrobacter* sp., and *Staphylococcus* sp. The only other upper atmosphere microbiology studies previously reporting *Micrococcus* sp. were from Imshenetsky et al. (1978) and Wainwright et al. (2003) who used a sounding rocket and balloon for collections, respectively. *Arthrobacter* sp. and *Staphylococcus* sp. have not appeared in upper atmosphere aerobiology literature, to our knowledge. Independent of culturing, we were able to identify *Pseudomonas stutzeri* as a significantly abundant OTU in Cruise samples. This was significant for two reasons: first*, P. stutzeri* has not appeared in other stratosphere microbiology studies; and second, no species-level identification of bacteria acquired from the stratosphere has been reported using culture-independent methods. To date, all stratosphere species identifications have been made using limited culture-based recoveries and subsequent Sanger sequencing with 16S. Encouragingly, our ABC system demonstrated that it can collect enough biomass in the stratosphere for DNA extraction and library preparation; and for the first time, culture-independent methods can be used to provide species-level resolution for bioaerosols above the Earth's troposphere.

We undertook extensive sampling of the C-20A aircraft itself to ensure a greater degree of confidence about *in situ* measurements. Based on 16S V4 sequencing data, our ground samples were clearly enriched in *Blastococcus* sp. BC412; *Georgenia* sp. JC82; *Modestobacter multiseptatus*; *Modestobacter versicolor*; *Modestobacter marinus*; *Clostridium sordellii*; *Ornithinimicrobium kibberense*; *Yaniella* sp. G5; *Nocardioides* sp. MSL 22; and *Blastococcus jejuensis*. These results identified the bacterial species abundant on the outside of the aircraft and, thus, potential influences on flight samples if free stream air models for our ABC probe's position were inaccurate. Many of the cultured isolates from ground samples were soil- and plant-associated *Bacillus* sp., including *B. carboniphilus, B. crescens, B. fumarioli, B. megaterium, B. aryabhattai, B. humi*, and *B. timonesis*. Other common soil bacteria *Bhargavaea ginseng* and *Streptomyces* sp. were detected on exterior portions of the aircraft, as well as *Gordonia paraffinivorans*, elsewhere associated with oil field environments (Xue et al., 2003). We did not determine species or strain level identities of Sanger sequenced isolates because of the short 466 bp region measured. Wider sequencing depth may have helped determine whether bacteria from ground samples were deposited during C-20A taxi time, take-off, landing, or maintenance and fueling operations. Significant changes between pre- and post-flight ground samples assayed over the same surfaces suggest that all aircraft probably shed microorganisms into the atmosphere and inherit new biomass as well (Pfaender and Swatek, 1970). But airplane traffic is probably a minor contributor to bioaerosols injected into the global atmosphere compared to more prevalent natural and human emission sources (Wéry et al., 2017).

We expected significant differences in the relative abundance and richness of bioaerosols owed to our study's wide spatial coverage across the US. Yet, OTUs acquired from spatially diverse flights seemed strikingly similar. The lack of regional patterns contradicts another continental scale study by Barberán et al. (2015) that acquired bioaerosols from dust samples at ground locations across the US. Because our study collected samples at higher altitudes, more mixing would be expected for longer-lived aerosol species. Other explanations for differences reported across the two studies might include seasonal influences, nearby emission sources and meteorological conditions. Longer-lasting studies using the C-20A would help reveal if bioaerosol concentrations shift during spring/summer months due to drier surface biomass getting aerosolized and stronger atmospheric convection patterns (Amato et al., 2017a; Wéry et al., 2017).

The incorporation of satellite data in our study offered supporting evidence for the homogenous distribution of bacterial taxa across separate flights. Both the MAIAC and MERRA-2 aerosol models showed similarities in the bulk type and abundance of atmospheric aerosols. One noteworthy exception was the CSAF flight that may have captured fire-related smoke plumes. The concentration of solvable SO_4_ was also higher on the CSAF flight compared to other days, suggesting the air mass sampled was drier. Precipitation is known to be associated with bioaerosol deposition (Deguillaume et al., 2008), so the relationship between regional smoke plumes, SO_4_, water vapor and the presence of bioaerosols warrants future investigation.

Even with statistical testing for significance and the removal of suspected contaminants, deciphering between taxa collected *in situ* and taxa associated with the aircraft/hardware or sample processing was challenging. Generally, flight samples yielded more reads and unique OTUs than measured in experimental controls, but filter blanks seated into the sampler inside the aircraft still resulted in numerous reads, including some totals that were higher than flight samples. For instance, *Staphylococcaceae* were commonly detected (especially dnOTU1), a bacterial family associated with human skin. Considering all sample filters were loaded into the cascade sampler inside the C-20A cabin, exposure to circulating air would be a likely source of background OTUs detected in our dataset. DNA-treated filters with small fragments of irradiated DNA and 16S sequencing reagents might also have contributed to a background signal. However, the primer set used for library preparation should not have amplified highly degraded DNA fragments. Tests of pristine filters and sample reagents resulted in no amplification of DNA using 16S qPCR reactions based on a standard curve derived from *Acinetobacter baumannii*. Thus, the source of baseline reads from negative controls were probably due to the influence of cabin air inside the C-20A. To address this baseline signal, we discarded OTUs from negative controls that had a higher mean in negative controls than in samples and yielded a mean relative abundance in negative controls > 1%.

Our pitot-style probe method of bioaerosol collection surely affected the type, size and number of bacteria captured in flight. For instance, biomass > 4 μm likely could not enter the probe. Some bioaerosols could have been lost inside the probe and inlet lines, too, though by mounting the hardware to the C-20A window plate we reduced the distance air traveled through the system. Overall, there remains a general need for standardized sampling methods in aerobiology because disparate collection techniques introduce variation and make horizontal comparisons difficult (Griffin et al., 2010; Amato et al., 2017b). Even an imperfect sampler used repeatedly by investigators sharing downstream methods could offer important insight into currently unknown aerobiology patterns. Historically, aerobiology studies using aircraft have relied upon culture-based recovery methods (with a bias toward heterotrophic plate counts) that underestimate the true quantity and diversity of bacteria in the upper atmosphere–this approach cannot identify slow-growing, unculturable, or inactivated bacteria, including fragmented cell components. Molecular methods address the limitation but do not readily distinguish between living and dead bacteria. Thus, a substantial portion of microbes detected by DNA sequencing might exist only as debris. In the spaceflight research community, a propidium monoazide (PMA) sequencing pre-treatment has been used as a viability marker to differentiate microbial sequences from dead cells vs. intact cells (Checinska et al., 2015). Future studies utilizing molecular methods might also consider standardizing primer sets and pursuing shotgun metagenomics assays in order to reduce bias associated with 16S reference databases which still contain many unclassified taxa. Our 16S V4 primers were based on the Earth Microbiome Project (Apprill et al., 2015; Parada et al., 2015; Walters et al., 2016) aimed at maximally inclusive coverage, but marine bacteria would still be under-detected due to repository biases.

By introducing the ABC, our hope is that other investigators can utilize the system for future aircraft studies or reproduce its basic components. All parts were easily manufactured or took advantage of relatively low cost commercially available products that can be obtained and installed on aircraft by follow-on investigator teams. For instance, the gelatinous filter we used was DNA-treated with gamma irradiation; often, field teams use sterile filters but such products still have a substantial DNA signal. Studies using other types of filters typically include aggressive methods to dislodge biomass (Smith et al., 2013), like vortexing or bead beating, introducing bias or damaging biomolecules (DeSantis et al., 2005). Another advantage of the gelatinous filter was that it could be dissolved in buffer, making the recovery of embedded bacteria 100% efficient for both culture-based and molecular methods. Because our flights were in arid regions and most of the sampling time was in the stratosphere with very low relative humidity, premature filter dissolving was not an issue for our system. In future years, we will pursue several modifications to the ABC system for improving bioaerosol capture and reducing system contaminants: (1) manufacturing a probe with a larger diameter to increase air flow rates above 8.5 l·min^−1^; (2) installing a valve at the probe opening to stop air from entering the inlet lines during take-off and landing; (3) building a glovebox on the C-20A workbench to accommodate the cascade sampler and prevent the cabin air from settling onto system surfaces; (4) creating a new filter membrane support grid from aluminum (not plastic) for easier pre-flight sterilization; and (5) recording system air volumes processed with a digitally-recorded flow meter. If combined with post-flight quantitative assays (e.g., cell counts though fluorescent microscopy or DNA abundance estimates with qPCR reactions), more precise air flow measurements would help establish global models of bioaerosol concentration, vertical distribution and atmospheric residence time.

It will always be difficult to determine bioaerosol origins given the tapestry of known emission sources and potential for long range atmospheric dispersal. A useful regional scale study might have sampling stations proximally-positioned to major aerosolization sources (e.g., wastewater treatment facilities, livestock feedlots). Downwind areas could then be sampled extensively with aircraft sweeps at multiple heights to make better correlations across time and space. Huffman and Santarpia (2017) discuss the importance of including “online” instruments on aircraft used for future aerobiology missions. Particle counters or LIDARs would be valuable for making real-time decisions in the air about where to sample and also for establishing stronger correlations between spectroscopic/fluorescent measurements and microorganisms identified later in the laboratory. Ultimately, studies should strive toward more routine and widespread flux measurements, analogous to other globally-tracked gaseous aerosol species (e.g., carbon dioxide, water vapor, ozone, nitrogen compounds) (Wéry et al., 2017).

Worldwide, bioaerosols are thought to represent 5–50% of atmospheric particles > 0.2 μm in diameter (Jaenicke, 2005; Després et al., 2012) and yet our current understanding of emission sources, transport history, and vertical distribution is quite rudimentary. Considering airborne biomass can travel thousands of kilometers over days if not weeks (Smith et al., 2013), atmospheric disease corridors likely exist (Prospero, 1999; Prospero et al., 2005), biodiversity impacts occur with microbes landing in new environments (Morris and Sands, 2017), and cloud chemistry and precipitation rates can be altered by cells (Delort et al., 2017; Hill et al., 2017), more coordinated, international research campaigns using aircraft in the troposphere and stratosphere should fly in future years. While addressing basic research questions about the dynamics of airborne microbes in the Earth's upper atmosphere, we can also gain knowledge about how to reliably collect and characterize trace levels of biomass present in extreme environments—such techniques will be relevant to astrobiology and the search for life on other worlds.

## Author contributions

DS led the study, designed the experiment and wrote the first draft of the manuscript. JDR and SJ performed the statistical analyses and compiled results from sequencing data. DG, PN, and SS did culture-based isolations, nucleic acid extractions and Sanger sequencing. KS extracted DNA for 16S V4 samples and conducted sequencing runs. HY and QT generated aerosol modeling data and analysis. TL, PM, AO, JL, SC, and JM provided engineering for the ABC design, instrument manufacturing and C-20A support. KI, LC, JT, and CJ reviewed the analysis, helped with data interpretation, and provided technical support. All authors contributed to reviewing, editing, and finalizing the manuscript.

### Conflict of interest statement

JDR, SJ, KS, KI, and LC are employees of Second Genome, Inc., a sequencing services company that produced the MiSeq data for this study. In exchange for paid services, they assisted with experiment planning and data generation. Reference herein to any specific commercial product, process, or service by trade name, trademark, manufacturer, or otherwise does not necessarily constitute or imply its endorsement, recommendation, or favoring by the United States government. The views and opinions expressed herein do not necessarily state or reflect those of the United States government and shall not be used for advertising or product endorsement purposes. The remaining authors declare that the research was conducted in the absence of any commercial or financial relationships that could be construed as a potential conflict of interest.

## Footnotes

^1^https://blast.ncbi.nlm.nih.gov/Blast.cgi.

^2^http://greengenes.secondgenome.com/downloads

^3^https://genelab.nasa.gov/

^4^https://tisch-env.com/product-literature/
